# Immune checkpoint inhibitor myocarditis: when profound troponin elevation diverges from unremarkable imaging

**DOI:** 10.1093/eschf/xvag029

**Published:** 2026-01-28

**Authors:** Roel S Driessen, P Stefan Biesbroek, Philippe J van Rosendael, Jeroen Slaats, Thijs M H Eijsvogels, Jasper L Selder, Roeland Lameris, Mariette Labots, Hans W Niessen, Marco J Götte

**Affiliations:** Department of Cardiology, Amsterdam UMC, location Vrije Universiteit, De Boelelaan 1117, 1081 HV Amsterdam, The Netherlands; Department of Cardiology, Amsterdam UMC, location Vrije Universiteit, De Boelelaan 1117, 1081 HV Amsterdam, The Netherlands; Department of Cardiology, Amsterdam UMC, location Vrije Universiteit, De Boelelaan 1117, 1081 HV Amsterdam, The Netherlands; Department of Clinical Chemistry, Amsterdam UMC, location Vrije Universiteit, Amsterdam, The Netherlands; Department of Medical BioSciences, Exercise Physiology Research Group, Radboud University Medical Center, Nijmegen, The Netherlands; Department of Cardiology, Amsterdam UMC, location Vrije Universiteit, De Boelelaan 1117, 1081 HV Amsterdam, The Netherlands; Department of Medical Oncology, Amsterdam UMC, location Vrije Universiteit, Amsterdam, The Netherlands; Department of Medical Oncology, Amsterdam UMC, location Vrije Universiteit, Amsterdam, The Netherlands; Department of Pathology, Amsterdam UMC, location Vrije Universiteit, Amsterdam, The Netherlands; Department of Cardiology, Amsterdam UMC, location Vrije Universiteit, De Boelelaan 1117, 1081 HV Amsterdam, The Netherlands

**Keywords:** Immune checkpoint inhibitor, Myocarditis, Cardiac troponin, Cardiac magnetic resonance imaging, Triple M syndrome

## Introduction

Myocarditis is a rare but severe immune-related adverse event (irAE) associated with immune checkpoint inhibitors (ICIs). These agents block inhibitory checkpoint molecules on T cells, such as programmed death (PD)-1, PD-L1, or CTLA-4, enhancing T-cell activation and boosting immune responses against tumours.^1^ Immune checkpoint inhibitors have rapidly emerged as a cornerstone in oncology, improving survival across various malignancies. However, their use is associated with a range of irAEs, including myocarditis. Although the incidence of ICI-associated myocarditis is reported to be <1%, mortality approaches 50%.^1,2^ Despite recently growing recognition, comprehensive data on this unpredictable condition remains limited. Here, we present a distinctive case of ICI-associated myocarditis with remarkable discrepancies between the clinical course, cardiac biomarkers, functional imaging, and histological findings. Based on our comprehensive analyses and known literature, we also provide a possible explanation for the apparent discrepancies.

## Case description

An 82-year-old male without prior cardiac disease was treated for Stage 3 melanoma with surgery and adjuvant dabrafenib/tramatenib without relevant side effects. After 2 years of follow-up, pulmonary metastases prompted initiation of anti-PD-1 ICI therapy with nivolumab. He had no recognized ICI-specific or traditional cardiovascular risk factors besides his age. Limited cardio-oncological evaluation, including history taking, physical examination, and electrocardiography (ECG), did not reveal any abnormalities. Therefore, he was not considered high risk or receiving cardioprotective medication, such as angiotensin-converting enzyme inhibitors, beta-blockers, or statins. Within 2 weeks, he developed rapidly progressive complaints of diplopia, ptosis, generalized weakness, and dyspnoea.

**Figure 1:**
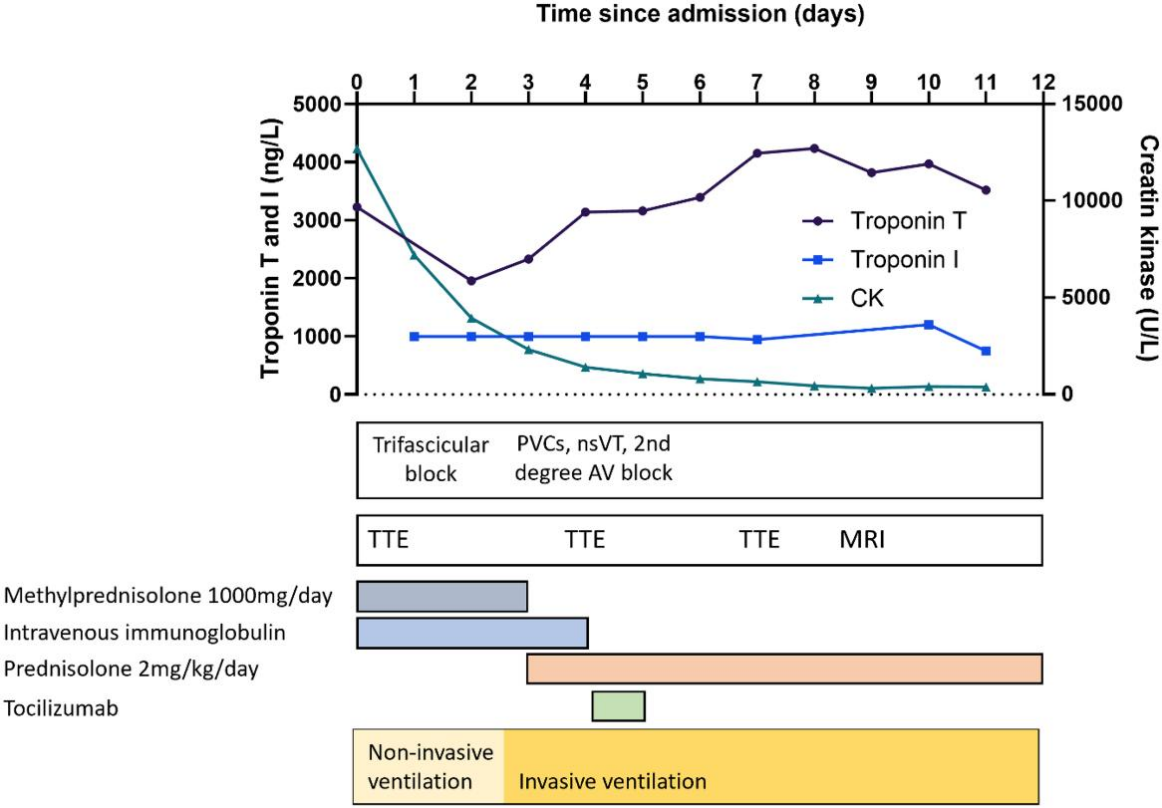
Timeline of diagnosis and management of patient with ICI-associated myocarditis. A timeline starting from the day of hospital admission, outlining the patient’s diagnostic progression, including biomarker changes, ECG abnormalities, and the timing of functional imaging, as well as treatment interventions, such as immunosuppressive therapy and ventilatory support. Cardiac troponin I upper limit of detection is 1000 ng/l except for the assessment on day 10 using another assay platform. AV block, atrioventricular block; CK, creatine kinase; ICI, immune checkpoint inhibitor; MRI, magnetic resonance imaging; nsVT, non-sustained ventricular tachycardia; PVCs, premature ventricular complexes; TTE, transthoracic echocardiogram

He presented to the emergency department with respiratory insufficiency without clear evidence of congestive heart failure, likely due to progressive muscle weakness. Electrocardiographic examination revealed a new first-degree atrioventricular (AV) block, right bundle branch block, and left anterior fascicular block. Laboratory evaluation demonstrated mildly elevated inflammatory markers but profoundly elevated muscular and cardiac biomarkers: creatine kinase (12 698 U/l; reference range <171 U/l), high-sensitivity cardiac troponin T (hs-cTnT; 3225 ng/l; reference range <14 ng/l), and hs-cTnI (>1000 ng/l; reference range <26 ng/l, upper limit of detection 1000 ng/l; *Figure 1*). Consistent with myositis, both aspartate aminotransferase (ASAT) and alanine aminotransferase (ALAT) were elevated, with ASAT > ALAT. Echocardiography showed preserved left ventricular ejection fraction (LVEF) with only mild inferior wall motion abnormalities. The diagnosis of ICI myocarditis was established according to the 2022 European Society of Cardiology Cardio-Oncology guidelines, based on elevated cardiac troponin (cTn) and multiple minor criteria, including clinical syndrome, new conduction system disorders, and other irAEs.^3^ Myasthenic crisis and myositis were diagnosed based on the clinical syndrome, neurologic examination, creatine kinase elevation, and positive acetylcholine receptor antibodies. The clinical diagnosis of triple M syndrome myocarditis, myositis, and myasthenic crisis was made.

Due to respiratory insufficiency, the patient was admitted to the intensive care unit and supported with non-invasive ventilation. Treatment was initiated with intravenous high-dose methylprednisolone (1000 mg/day for 3 days) and immunoglobulin to a total dose of 2 g/kg over 5 days.

**Figure 2:**
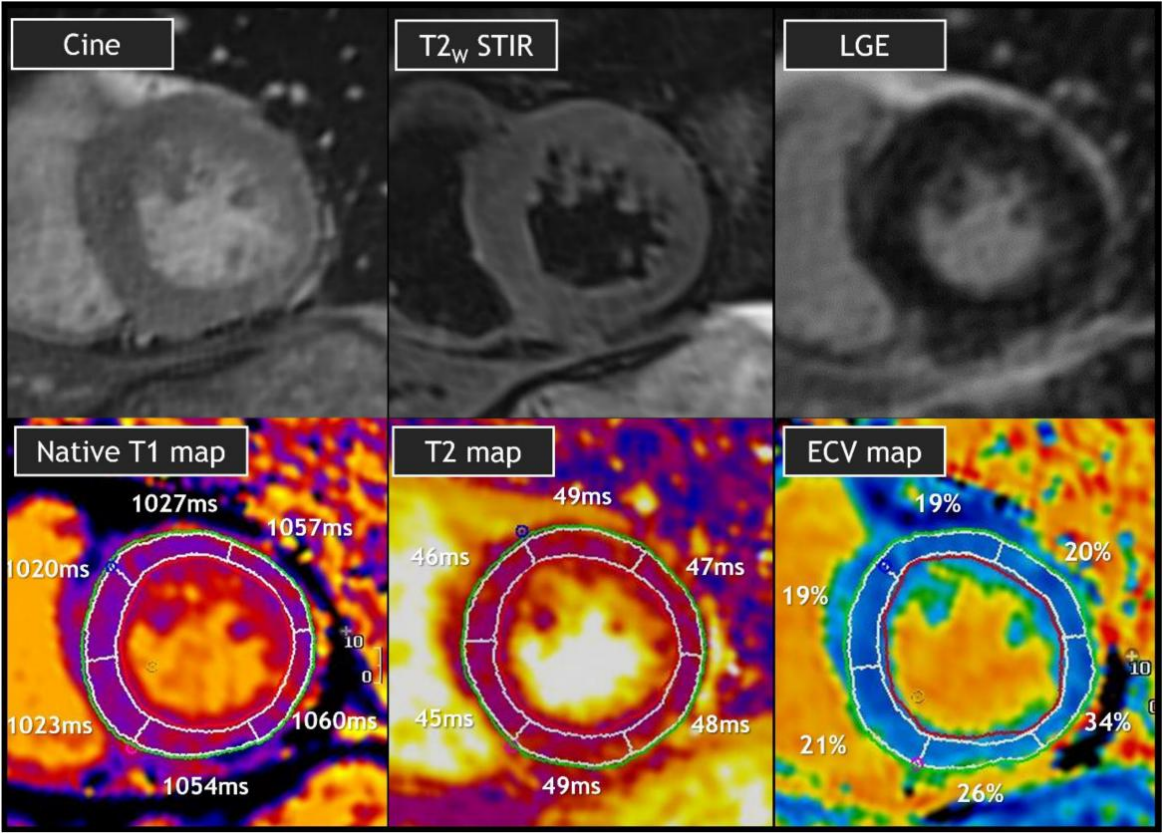
MRI findings. Exemplary MRI images showing normal to slightly increased left ventricular wall thickness on cine image, no clear evidence of focal oedema on T2w STIR image, and no clear focal fibrosis on LGE image. Furthermore, quantitative parametric MRI images neither showed clear evidence of global fibrosis with normal to borderline elevated T1 values (mean 1026 ms, normal <1052 ms) and ECV values (mean 22%, normal 20%–29%), nor evidence of global oedema with normal to borderline elevated T2 values (mean 47 ms, normal values <50.3 ms). ECV, extracellular volume; LGE, late gadolinium enhancement; ICI, immune checkpoint inhibitor, late gadolinium enhancement; T2w STIR, T2-weighted short tau inversion recovery

Initially, the patient improved with reduced respiratory support and a modest decline in cTn concentrations. On Day 3, respiratory insufficiency recurred with cardiac arrhythmias, including frequent premature ventricular contractions, non-sustained ventricular tachycardias, and paroxysmal second-degree AV block. Repeat echocardiography continued to demonstrate a preserved LVEF, but a slightly reduced global longitudinal strain (GLS) of −16.8%. The patient was intubated and interleukin-6 receptor inhibitor tocilizumab was added. High-dose corticosteroids were continued (2 mg/kg/day). Despite intensive treatment, hs-cTnT levels remained persistently elevated (3000–4000 ng/L over a 12-day period), while creatine kinase levels rapidly declined. Because of the discrepancy between biomarker levels of myocardial injury and preserved function, cardiac magnetic resonance imaging (MRI) was performed on Day 9. Remarkably, MRI showed normal LVEF (61%), no clear global or focal oedema or fibrosis; there was no apparent late gadolinium enhancement (LGE), and quantitative T1, T2, and extracellular volume (ECV) assessments were all normal with only borderline elevations in the inferior and inferolateral wall (*Figure 2*).

**Figure 3:**
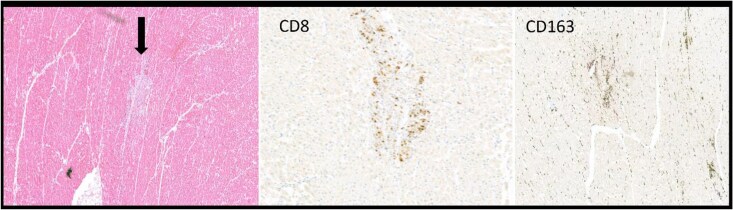
Pathology findings. Microscopic pathology images showing a small focus of inflammatory infiltrate (black arrow) with positive staining for CD8 and CD163. This is typically consistent with ICI-mediated myocarditis. ICI, immune checkpoint inhibitor

Despite aggressive therapy, the patient failed to recover, primarily due to diaphragmatic weakness that precluded spontaneous breathing. In the setting of advanced age, multiorgan failure, limited prospects for meaningful recovery, and in accordance with the patient’s wishes, active treatment including mechanical ventilation was discontinued. For the same reasons, no additional experimental but promising immunomodulatory therapies, such as abatacept or plasma exchange were initiated, given the low likelihood of benefit beyond already administered first-line and second-line therapy. On Day 12, the patient died of respiratory insufficiency. Post-mortem examination confirmed myocarditis with areas of decreased nitroblue tetrazolium staining that were partly homogeneous and partly patchy. This was somewhat unexpected regarding the cardiac MRI findings. Multiple small foci of inflammatory cell infiltrates, not diffusely apparent, showed positive staining for CD8 (cytotoxic T lymphocytes), CD45 (leucocytes), CD3 (T lymphocytes), and CD163 (M2 macrophages), which is compatible with ICI-induced myocarditis. Within these areas increased the expression of PD-L1 was detected on cardiomyocytes’ plasma membrane (*Figure 3*). Herein also necrotic cardiomyocytes (C3d positivity), granulation tissue, and minor fibrosis were found. The estimated area of cell death was only 0.44%, of which half showed necrosis and the other half granulation tissue. These findings are consistent with immune-mediated myocarditis. Skeletal muscles showed myositis, including the diaphragm and psoas. Micrometastases of melanoma were identified in the lungs.

## Discussion

This case of ICI-induced myocarditis demonstrates a remarkable and clinically significant mismatch: markedly elevated cTn concentrations alongside preserved LVEF and normal cardiac MRI findings. To our knowledge, the degree of biomarker-imaging discordance described here has not been documented. Such discrepancies complicate diagnosis and management, as profoundly elevated cTn usually correlates with myocardial injury, functional impairment, and possibly overt heart failure.

Hs-cTnT and hs-cTnI concentrations were strikingly elevated for nearly 2 weeks, typically indicating myocardial damage detectable on imaging. However, neither ventricular dysfunction nor abnormal MRI findings were present, which is highly unusual compared with previous reports. A recent registry by Van den Berg et al. reported that 16% of patients receiving ICI therapy developed a significant rise in hs-cTnT, yet only 31% of these were ultimately diagnosed with ICI myocarditis. Median hs-cTnT levels among established myocarditis cases were 242 ng/l [interquartile range (IQR) 168–1109], compared with 41 ng/l (IQR 36–84) in patients without signs of myocarditis or cardiac dysfunction.^4^ Although elevated, these values remain far below the persistently extreme levels (3000–4000 ng/l) observed in our patient. It is now recognized that in muscle diseases, such as myositis, cTnT may be falsely elevated due to re-expression in skeletal muscles.^5^ This is especially relevant in ICI-myocarditis cases due to the common overlap with neuromuscular diseases in up to 25% of patients. In the presented case, however, both cTnT and cTnI were markedly elevated, excluding ectopic cTnT release. Potential causes of analytical interference were carefully evaluated. cTnI concentrations were cross-checked using an alternative assay platform, and PEG-precipitation experiments ruled out macrotroponin formation due to circulating autoantibodies binding to cTn and fibrin clots. These observations, next to clinical findings such as the ECG abnormalities, supported the authenticity of the biomarker findings.

One possible explanation for the observed discrepancy is increased cardiomyocyte membrane permeability induced by inflammation. This can lead to leakage of cTnT and cTnI particles through cell wounds, membranous blebs, and extracellular vesicles. Approximately 5% of total cTn exists in a freely soluble cytoplasmic fraction that undergoes daily turnover.^6^ Leakage of this fraction could yield markedly elevated cTn concentrations without a significant loss of functional cardiomyocytes. This is consistent with histopathological findings of small foci of inflammation that were unlikely to be detected on cardiac MRI-derived LGE images or locally abnormal T1, T2, and ECV values. This mechanism is also thought to explain, at least in part, elevated cTn concentrations seen in athletes following strenuous exercise.^7^

Cardiac MRI is a unique, well-recognized imaging modality for non-invasive myocarditis diagnosis, applying the updated Lake Louise Criteria based on T1/T2 mapping and LGE.^8^ In infectious myocarditis, sensitivity and specificity are high. However, recent series indicate that these criteria may be less reliable in ICI myocarditis, raising questions about their diagnostic applicability in this setting. A multicentre study by Thavendiranathan et al. reported a sensitivity of only 48% when applying Lake Louise Criteria. This low sensitivity was primarily driven by the low appearance of myocardial oedema criteria (53%).^9^ Similarly, Lerchner et al. recently reported LGE in 56% and abnormal T2 mapping in only 8% of cases.^10^ Notably, hs-cTnI levels in the latter cohort were relatively low (median 43 ng/l), which could at least partly explain cardiac MRIs low sensitivity. In contrast, our patient demonstrated persistently and markedly elevated hs-cTnI levels (>1000 for days). Yet cardiac MRI findings remained normal, illustrating an even more pronounced biomarker-imaging mismatch. Even when advanced cardiac MRI protocols including T1 and T2 mapping are used, sensitivity remains suboptimal. Although MRI sensitivity is thought to be reduced during the very acute phase of myocarditis, the delayed MRI (Day 9) and prior immunosuppressive therapy in our case may have influenced detectable inflammatory signals such as abnormal T1/T2 mapping. A relatively simple GLS assessment with echocardiography, however, showed abnormal results in the present case and could perhaps serve as an alternative, more sensitive tool in diagnosing ICI myocarditis. Nevertheless, the discrepancies between clinical and imaging results in the present case highlight the potential value of endomyocardial biopsy (EMB). While EMB remains the diagnostic gold standard, it could also serve as a reliable alternative diagnostic strategy. Sampling error and procedural risks restrict routine use, but in cases with discordant findings, earlier biopsy may be justified. Our case illustrates that definitive diagnosis was only achieved post-mortem; had EMB been pursued during admission, histology might have clarified the paradox earlier. Future diagnostic algorithms may need to place greater emphasis on EMB in selected ICI-myocarditis cases.

The clinical implications of isolated or disproportionate cTn elevations under ICI therapy are profound. Currently, European Society of Cardiology guidelines recommend immediate cessation of ICI therapy and prompt initiation of high-dose corticosteroids once myocarditis is suspected.^3^ Yet, our case demonstrates that cTn elevations may persist in the absence of functional compromise, raising uncertainty about whether aggressive immunosuppression is always warranted. Clinicians face a dilemma: withholding oncological therapy risks tumour progression, while continuation risks fatal cardiac toxicity. Prospective data are lacking, but multidisciplinary teams must weigh biomarker dynamics, imaging, and patient trajectory in individualized decisions. The possibility of safely rechallenging patients with ICI after isolated troponin elevation remains an open and pressing question for cardio-oncology practice. More broadly, this case underscores the need for nuanced interpretation of biomarkers and imaging in ICI myocarditis. Rather than applying paradigms from viral myocarditis, clinicians may need to adopt ICI-specific thresholds and criteria, recognizing that small histological lesions can drive major biomarker elevations without overt dysfunction. Registries and collaborative studies should prioritize systematic integration of biomarker, imaging, biopsy, and clinical outcome data to refine diagnostic algorithms.

In conclusion, ICI-induced myocarditis may present with seemingly disproportionately elevated cardiac biomarkers despite preserved LVEF and normal cardiac MRI findings. The clinical interpretation of elevated cTn concentrations and relatively normal echocardiography and cardiac MRI remains complex. Perhaps we should interpret these biomarkers and cardiac MRI differently in ICI-induced myocarditis than in other clinical scenarios. This case highlights the complexity of ICI-myocarditis diagnosis and management, emphasizing the need for multidisciplinary care and further research.

## Declarations

### Data Availability

The data underlying this article will be shared on reasonable request to the corresponding author.
